# Associations between soil-transmitted helminthiasis and viral, bacterial, and protozoal enteroinfections: a cross-sectional study in rural Laos

**DOI:** 10.1186/s13071-019-3471-2

**Published:** 2019-05-07

**Authors:** Anna N. Chard, Kelly K. Baker, Kevin Tsai, Karen Levy, Jeticia R. Sistrunk, Howard H. Chang, Matthew C. Freeman

**Affiliations:** 1 Department of Environmental Health, Rollins School of Public Health, Emory University, Atlanta, Georgia 30322 USA; 2 Department of Occupational and Environmental Health, College of Public Health, University of Iowa, Iowa City, Iowa 52242 USA; 3 Department of Biostatistics and Bioinformatics, Rollins School of Public Health, Emory University, Atlanta, Georgia 30322 USA

**Keywords:** Soil-transmitted helminths (STH), Microparasite, Enteric disease, Co-infection Water, sanitation, hygiene (WASH), qPCR

## Abstract

**Background:**

Humans are susceptible to over 1400 pathogens. Co-infection by multiple pathogens is common, and can result in a range of neutral, facilitative, or antagonistic interactions within the host. Soil-transmitted helminths (STH) are powerful immunomodulators, but evidence of the effect of STH infection on the direction and magnitude of concurrent enteric microparasite infections is mixed.

**Methods:**

We collected fecal samples from 891 randomly selected children and adults in rural Laos. Samples were analyzed for 5 STH species, 6 viruses, 9 bacteria, and 5 protozoa using a quantitative reverse transcription polymerase chain reaction (qRT-PCR) assay. We utilized logistic regression, controlling for demographics and household water, sanitation, and hygiene access, to examine the effect of STH infection on concurrent viral, bacterial, and protozoal infection.

**Results:**

We found that STH infection was associated with lower odds of concurrent viral infection [odds ratio (OR): 0.48, 95% confidence interval (CI): 0.28–0.83], but higher odds of concurrent bacterial infections (OR: 1.81, 95% CI: 1.06–3.07) and concurrent protozoal infections (OR: 1.50, 95% CI: 0.95–2.37). Trends were consistent across STH species.

**Conclusions:**

The impact of STH on odds of concurrent microparasite co-infection may differ by microparasite taxa, whereby STH infection was negatively associated with viral infections but positively associated with bacterial and protozoal infections. Results suggest that efforts to reduce STH through preventive chemotherapy could have a spillover effect on microparasite infections, though the extent of this impact requires additional study. The associations between STH and concurrent microparasite infection may reflect a reverse effect due to the cross-sectional study design. Additional research is needed to elucidate the exact mechanism of the immunomodulatory effects of STH on concurrent enteric microparasite infection.

**Electronic supplementary material:**

The online version of this article (10.1186/s13071-019-3471-2) contains supplementary material, which is available to authorized users.

## Background

Humans are susceptible to over 1400 known parasite species, including viruses, bacteria, protozoa, helminths and fungi [1]. Co-infection by multiple pathogens is common and is often considered the rule rather than the exception among populations living in socially and economically marginalized communities, rural areas, and tropical or subtropical climate zones [2]. Co-infections result in a range of neutral, facilitative, or antagonistic interactions [3, 4]. These interactions have important implications for host susceptibility to infection, disease severity [3, 4], and treatment efficacy [5–7].

Soil-transmitted helminth (STH) infections are one of the most ubiquitous human infections, affecting over one billion people worldwide [8, 9]. It is estimated that STH co-infections occur in over 800 million people [10]. However, interactions between STH and microparasites (defined here as a virus, bacteria, or protozoa) within the human host and the impacts of these interactions on human health are poorly understood [11].

Helminths are powerful immunomodulators [12, 13] and can affect microparasite infections via at least two distinct immune mechanisms. First, helminths usually induce a type 2 (Th2) immune response, including elevations in cytokines such as interleukin 4 (IL-4), IL-5, and IL-13, as well as development of Th2 helper T cells [11, 14, 15]. Microparasites generally induce a type 1 (Th1) immune response, which elevates cytokines IL-12, IL-17, IL-23, interferon-γ (IFN-γ) and tumor necrosis factor (TNF)-α [11, 14]. The Th2 cytokines downregulate the Th1 cytokines that enable hosts to fight microparasite infection, resulting in a dampened immune response [14]. Secondly, to protect themselves from host immunity, helminths, like microparasites, suppress both Th1 and Th2 responses by enhancing regulatory T cell (T_reg_) activity, which causes the release of regulatory cytokines such as IL-10 and transforming growth factor (TGF)-β, and leads to reduced immune responses against microparasite infection [15]. Helminths may also interact with microparasites via shared resources [13, 16, 17] by, for example, reducing the surface area availability for microparasite attachment or by monopolizing a cell type necessary for microparasite replication [18]. Such disparate responses may lead to within-host interactions by altering host susceptibility to infection [11, 19], altering the virulence of co-infecting pathogens [11, 19], and affecting the host’s ability to clear co-infecting pathogens [19, 20].

Understanding the impact of pathogen co-infection on human health is difficult due to the diversity of co-infecting species and their numerous possible interactions [16]. Even though many humans typically harbor multiple pathogens [15], most studies of co-infection measure interactions between pairs of parasites [16]. In this study, we examine co-infection between five STH species and 20 microparasites, including six viruses, nine bacteria, and five protozoa in human hosts. To identify trends in pathogen interaction, we evaluate interspecific associations between STH and enteric microparasite infection at the at the taxa level (e.g. viruses, bacteria, and protozoa).

## Methods

### Study setting and design

This cross-sectional study was nested within the Water, Sanitation, and Hygiene for Health and Education in Laotian Primary Schools (WASH HELPS) study, a longitudinal cluster-randomized trial evaluating a comprehensive school-based water, sanitation, and hygiene (WASH) intervention in 100 schools in Saravane Province, Lao People’s Democratic Republic (Lao PDR; Laos). Detailed methods of the parent study are described elsewhere [21]. The WASH HELPS study is registered at clinicaltrials.gov (NCT02342860).

Of the 100 schools participating in the WASH HELPS study, 50 (25 intervention and 25 comparison) were selected using stratified random sampling based on district size and WASH HELPS study intervention status. In each school-hosting village (there is only one school per village), we randomly selected 25 households. Households were eligible for inclusion if they had a child attending the primary school participating in the WASH HELPS study, and that pupil had a sibling < 5 years-old living in the household. At each household, the female head of household was surveyed on household demographics, asset and animal ownership, recent illness among household members, and WASH access and behaviors. Structured observations of WASH facilities were made when available.

In conjunction with the household survey, we collected stool samples from the pupil, the pupil’s parent/caregiver (preference was given to female parent/caregiver), and the pupil’s sibling < 5 years-old (if there were multiple siblings, preference was given to youngest sibling). To collect the stool samples, the female parent/caregiver was given three pre-labeled, resealable plastic bags each containing a plastic spoon. Caregivers were given diapers to collect stool from infants, when applicable. Written and pictorial instructions for stool collection were printed on the plastic bag, and participants were also provided verbal instructions. Participants were instructed to collect their first morning stool, and were informed that the field team would return to the household the following morning to collect all samples. If households did not return all three stool samples on the first day, participants were reminded of the stool collection procedures, provided new bags and spoons if needed, and a second return visit was made the following day. Stool samples were transported with a cold chain to the field laboratory within two hours of collection.

Upon collection, all samples were tested for STH using the Kato-Katz method [22]. For this sub-study, stool samples from a subset of 297 households were randomly selected for additional enteropathogen analysis *via* quantitative reverse transcription polymerase chain reaction (qRT-PCR). Households were eligible for inclusion in this sub-study only if all three subjects in the household (adult, school-aged child, and child < 5 years-old) returned their stool sample on the same day. Households were randomly selected, proportional to district size, village size, and WASH HELPS intervention status, from households participating in the household survey and STH testing by Kato-Katz.

All data were collected between February-April 2017 (dry season), prior to annual school-based preventative chemotherapy (PC) for STH. The time frame corresponded with the final round of data collection and conclusion of the WASH HELPS study [21].

### Laboratory analysis

Following analysis for STH *via* Kato-Katz, 200 mg of stool was aliquoted into a DNA/RNA Shield Collection and Lysis Tube (Zymo Research, Irvine, CA, USA) containing a lysis buffer and bead beating system, and beaten for 20 min using a Disrupter Genie vortexer (Scientific Industries, Bohemia, NY, USA) [23]. One field control was processed each day using DNA/RNA-free water to evaluate the possibility of false positives from contamination in the field laboratory during sampling. Samples were kept frozen at − 20 °C until transported to a laboratory at Emory University, where they were subsequently stored at − 80 °C until extraction.

Total nucleic acid was extracted from samples using the ZymoBIOMICS DNA/RNA Mini Kit (Zymo Research, Irvine, CA, USA), according to manufacturer instructions. Samples were spiked with bacterophage MS2 (ZeptoMetrix, Buffalo, NY, USA), an external control, to monitor extraction and amplification efficiency [23]. One extraction blank was included per batch to exclude the possibility of false positives from contamination during extraction. Extractions were stored at − 80 °C until transported on dry ice to the University of Iowa for qRT-PCR analysis.

We created a custom TaqMan Array Card (TAC) (Thermo Fisher, Carlsblad, CA, USA) with compartmentalized, probe-based qPCR assays for 25 enteropathogens, including: five STH (*Ancylostoma duodenale*, *Ascaris lumbricoides*, *Necator americanus*, *Strongyloides stercoralis* and *Trichuris trichiura*); six viruses (astrovirus, adenovirus, norovirus GI, norovirus GII, rotavirus, sapovirus); nine bacteria (*Aeromonas* spp., *Campylobacter jejuni*, *Clostridium difficile*, enteroaggregative *Escherichia coli* (EAEC), enterohemorrhagic *E. coli* (EHEC), atypical or typical enteropathogenic *E. coli* (EPEC), heat-labile- (LT) or heat-stable (ST) enterotoxigenic *E. coli* (ETEC), *Salmonella enterica* and *Shigella* spp./Enteroinvasive *E. coli* (EIEC); and five protozoa (*Cryptosporidium* spp., *Cryptosporidium hominus*, *Cryptosporidium parvum*, *Entamoeba histolytica* and *Giardia intestinalis*) [24, 25]. The TAC included probes for the MS2 external control, as well as an 18S rRNA internal control. The TAC primer and probe sequences are listed in Additional file 1: Table S1.

TAC preparation was prepared based on the protocol described by Liu et al. [24]. Ag-Path-ID One-Step RT-PCR kit (Thermo Fisher, Waltham, MA) was used as the master mix reagent for the TAC analysis. Bovine serum albumin (BSA) was also applied into the TAC master-mix to prevent the possibility of PCR inhibition that may arise in nucleic acids extracted from stools [26, 27]. For each sample, 40 µl of DNA/RNA extract of equal volumes of DNA and RNA was mixed with 50 µl of 2× RT-buffer, 4 µl of 25× AgPath enzyme, 5.4 µl of nucleic acid-free water, and 0.6 µl of 50 mg/ml BSA to a total volume of 100 µl. All TAC runs were completed in a ViiA7 instrument with QuantStudio 7 software (Thermo Fisher, Waltham, MA), and the cycling conditions were as follows: holding stages of 45 °C for 20 min and 95 °C for 10 min, followed by 45 cycles of 95 °C for 15 s and 60 °C for 1 min.

TAC data were manually read by two independent researchers. True amplification was validated by inspecting the multicomponent plot for increases in fluoresce for the FAM-based gene-specific probe. Conflicting results were resolved by a third independent researcher. Samples were considered positive only when the corresponding field and extraction blanks were negative, otherwise the data were considered invalid [25].

### Measures

Adult participants reported the age and sex of themselves, their primary school-aged child, and their child under five years-old. The following variables were reported by the female head of household: household ethnicity, in which households of non-Lao-Tai ethnicity were considered ethnic minorities; the number of household members, which was derived by listing and counting all people currently living in the household full time; animal ownership, which was defined as owning any cows, goats, sheep, poultry (chickens or ducks), or pigs; and the main source of household drinking water, which was further classified as improved/unimproved according to the World Health Organization/United Nations International Children’s Fund (WHO/UNICEF) Joint Monitoring Programme (JMP) standard definitions [28].

The following variables were reported by enumerators using structured observation: the presence of a household toilet, which was further classified as improved/unimproved according to WHO/UNICEF JMP standard definitions [28]; and the presence of a household basic handwashing facility, classified according to WHO/JMP standard definitions as having soap and water [28]. Socioeconomic status was determined through a series of questions and observations about household construction materials (roof, floor, and walls), ownership of a mobile phone, and presence of electricity. These variables were chosen based on those used in the Demographic and Health Surveys for measures of wealth in Laos [29]. We used principal components analysis to derive one single wealth metric from all of the wealth assets combined [30].

*Escherichia coli* pathotypes were classified according to the following gene targets: EAEC (*aatA* and/or *aaiC*), EHEC (*eae* with *stx1* and/or *stx2*, and without *bfpA*), typical EPEC (*bfpA* with or without *eae*), atypical EPEC (*eae* without *bfpA*, *stx1*, or *stx2*), ETEC (*eltB* for heat-labile toxin [LT] and *estA* with or without *eltB* for heat-stable toxin [ST]) [31]. The number of microparasite infections was derived by summing all positive pathogens (range: zero to 20). We chose to use the *ipaH* gene target to be consistent with approaches used in other recent enteric disease studies of under-five children. However, *ipaH* occurs in *Shigella* spp. and EIEC, and does not validate the presence of the large virulence plasmid of other virulence genes that are unique to *Shigella* spp.

### Statistical analysis

All data were analyzed using Stata Statistical Software: Release 15 (StataCorpLP, College Station, TX, USA).

We estimated the odds of concurrent microparasite infection using three separate logistic regression models for viral, bacterial and protozoal infection outcomes. For the primary analysis, the main exposure of interest was any STH infection, as determined by qRT-PCR detection. Secondary analyses examined specific STH species (i.e. hookworm, *A. lumbricoides*, *T. trichiura* or *S. stercoralis*) as main predictors. We controlled for the presence of the non-outcome microparasite taxa (e.g. the model of the association between STH and viral infection also controlled for concurrent bacterial and protozoal infection), as well as the following covariates determined *a priori* based on biological plausibility of affecting odds of both outcomes and STH infection: age group (i.e. adult, school-aged child, child < 5 years-old), sex, socioeconomic status, ethnic minority status, household population size, improved household toilet, improved household drinking water source, basic household handwashing facility, household animal ownership, and whether the school in the village was a beneficiary of a UNICEF WASH in Schools intervention. Random intercepts were included at the village and household levels to account for clustering.

The associations between STH infection or STH species and the number of concurrent microparasite infections were determined using separate Poisson regression models and are reported as beta coefficients representing the change in number of microparasite infections among subjects with STH (or specific STH species) infection compared to those without. Models included random intercepts at the village and household levels, and included the same covariates as the logistic regression models.

All models were assessed for effect modification by age group. All analyses were evaluated for statistical significance at *P* < 0.05.

## Results

We collected a total of 2269 fecal samples from the same number of participants. Of these, 891 participants from 297 households were eligible for inclusion in this study because all three participants in the selected household (adult, school-aged child, and child < 5 years-old) returned their stool sample on the same day. Data from 746 participants were included in the analysis [*n *= 1 excluded due to insufficient sample amount for nucleic acid extraction; *n *= 144 excluded due to suspected field (*n *= 66) or laboratory (*n *= 78) contamination of one or more target pathogens]. The study population is described in Table 1.

**Table 1:** Description of study population, Saravane Province, Laos, 2017

Characteristics	Total (*N *= 746)	Child < 5 years (*N *= 249)	School-aged child (*N *= 247)	Adult (*N *= 250)
Female, *n* (%)	496 (66.5)	121 (48.6)	125 (50.6)	250 (100)
Median (IQR^a^) age (yrs)	9 (24.5)	4 (2.0)	9 (3.0)	32 (11.0)
Ethnic minority^b^, *n* (%)	351 (47.1)	119 (47.8)	114 (46.2)	118 (47.2)
Household has improved toilet^c^, *n* (%)	230 (30.1)	75 (30.1)	77 (31.2)	78 (31.2)
Household utilizes improved drinking water source^c^, *n* (%)	355 (47.6)	118 (47.4)	117 (47.4)	120 (48.0)
Household has basic handwashing facility^c^, *n* (%)	262 (35.1)	87 (34.9)	89 (36.0)	86 (34.4)
Median (IQR) number of people living in household	6.5 (4)	6 (4)	6 (4)	7 (4)
Household owns animals, *n* (%)	713 (95.6)	238 (95.6)	236 (95.6)	239 (95.6)
Beneficiary of school WASH^b^ intervention, *n* (%)	374 (50.1)	126 (50.6)	122 (49.4)	126 (50.4)

Values for household-level covariates vary across age groups due to exclusion of some samples for suspected contamination

^a^Defined as those not belonging to the Lao-Tai ethnic group

^b^Interquartile range (IQR); water, sanitation, and hygiene (WASH)

^c^Classified according to WHO/UNICEF Joint Monitoring Programme standard definitions [28]

At least one STH was present in 61.3% of participants (Table 2); hookworm was the most prevalent STH infection (43.6%). Of the microparasites, bacterial infections were the most common (86.8%), followed by protozoal infections (72.8%), then viral infections (33.2%). Prevalence of individual microparasites are described in Table 2. EAEC was the most common bacterial infection (47.3%), *Giardia* was the most common protozoal infection (68.9%), and rotavirus was the most common viral infection (24.1%). Kato-Katz results are presented in Additional file 2: Figure S1.

**Table 2:** Prevalence of soil-transmitted helminth (STH), viral, bacterial, and protozoal infections, Saravane Province, Laos, 2017

Infections	Total (*N *= 746)	Child < 5 years (*N *= 249)	School-aged child (*N *= 247)	Adult (*N *= 250)
	*n* (%)	*n* (%)	*n* (%)	*n* (%)
Any STH^a^	457 (61.3)	133 (53.4)	154 (62.4)	170 (68.0)
Hookworm	325 (43.6)	81 (32.5)	118 (47.8)	126 (50.4)
*A. lumbricoides*	61 (8.2)	23 (9.2)	19 (7.7)	19 (7.6)
*T. trichiura*	119 (16.0)	40 (16.1)	46 (18.6)	33 (13.2)
*S. stercoralis*	154 (20.6)	38 (15.3)	45 (18.2)	71 (28.4)
Any virus^b^	248 (33.2)	90 (36.1)	78 (31.6)	80 (32.0)
Astrovirus	18 (2.4)	8 (3.2)	6 (2.4)	4 (2.0)
Adenovirus	6 (0.8)	0 (0.0)	4 (1.6)	2 (0.8)
Norovirus GI	8 (1.2)	1 (0.4)	4 (1.6)	3 (1.2)
Norovirus GII	55 (7.4)	21 (8.4)	15 (6.1)	19 (7.6)
Rotavirus	180 (24.1)	61 (24.5)	58 (23.5)	61 (24.4)
Sapovirus	11 (1.5)	8 (3.2)	3 (1.2)	0 (0)
Any bacteria^c^	640 (86.8)	216 (86.8)	206 (83.4)	218 (87.2)
*Aeromonas* spp.	224 (30.0)	54 (21.7)	70 (28.3)	100 (40.0)
*Clostridium difficile*	8 (1.1)	3 (1.2)	3 (1.2)	2 (0.8)
*Campylobacter jejuni*	163 (21.9)	69 (27.7)	55 (22.3)	39 (15.6)
EAEC	353 (47.3)	113 (45.4)	109 (44.1)	131 (52.4)
EHEC	91 (12.2)	21 (8.4)	29 (11.7)	41 (16.4)
EPEC	262 (35.1)	94 (37.8)	91 (36.8)	77 (30.8)
Typical	60 (8.1)	15 (6.0)	20 (8.1)	25 (10.0)
Atypical	202 (27.1)	79 (31.7)	71 (28.7)	52 (20.8)
ETEC	278 (37.3)	95 (38.2)	70 (28.3)	113 (45.2)
LT-ETEC	78 (10.5)	35 (14.1)	13 (5.3)	30 (12.0)
ST-ETEC	200 (26.8)	60 (24.1)	57 (23.1)	83 (33.2)
*Shigella* spp./EIEC	117 (15.7)	37 (14.9)	36 (14.6)	44 (17.6)
*Salmonella enterica*	37 (5.0)	8 (3.2)	9 (3.6)	20 (8.0)
Any protozoa^d^	543 (72.8)	203 (81.5)	188 (76.1)	152 (60.8)
*Cryptosporidium* spp.	105 (14.1)	41 (16.5)	34 (13.8)	30 (12.0)
* Cryptosporidium hominus*	1 (0.1)	0 (0)	1 (0.4)	0 (0)
*Cryptosporidium parvum*	0 (0)	0 (0)	0 (0)	0 (0)
*Entamoeba histolytica*	1 (0.1)	1 (0.4)	0 (0)	0 (0)
*Giardia intestinalis*	514 (68.9)	195 (78.3)	179 (72.5)	140 (56.0)
Mean (standard deviation) number of microparasites	3.3 (1.7)	3.3 (1.5)	3.1 (1.7)	3.3 (1.8)

^a^Soil-transmitted helminth (STH) includes one or more of the following helminths: hookworm (*N. americanus* and/or *A. duodenale*)*, A. lumbricoides*, *T. trichiura*, or *S. stercoralis*

^b^Virus includes one or more of the following pathogens: astrovirus, adenovirus, norovirus GI, norovirus GII, rotavirus, or sapovirus

^c^Bacteria includes one or more of the following pathogens: *Aeromonas*, *C. difficile*, *C. jejuni*, EAEC, EHEC, EPEC (typical or atypical), LT- or ST-ETEC, *Shigella* spp./Enteroinvasive *E. coli*, or *Salmonella*

^d^Protozoa includes one or more of the following pathogens: non-hominus and non-parvum *Cryptosporidium* spp., *C. hominus*, *C. parvum*, *E. histolytica*, and *G. intestinalis*

All data come from quantitative reverse transcription polymerase chain reaction (qRT-PCR) analysis

Associations between STH infection and viral, bacterial, and protozoal infection are described in Table 3. Age was not a significant effect modifier for any primary or secondary outcomes, so we present unstratified results. STH infections were associated with lower odds of concurrent viral infection; this trend was consistent across all STH species and was statistically significant for any STH infection [odds ratio (OR) = 0.48, 95% confidence interval (CI): 0.28–0.83] and *S. stercoralis* (OR= 0.52, 95% CI: 0.29–0.95). STH infections were associated with higher odds of concurrent bacterial infection. This trend was statistically significant for any STH infection (OR= 1.81, 95% CI: 1.06–3.07) and *T. trichiura* (OR= 5.97, 95% CI: 2.05–17.40). STH infections were associated with higher odds of concurrent protozoal infection; this trend was consistent across all STH species and was statistically significant for hookworm (OR= 1.78, 95% CI: 1.11–2.84).

**Table 3:** Associations between STH infection and concurrent virus, bacteria, protozoa infection, and number of microparasite infections, Saravane Province, Laos, 2017 (*n *= 746)

	Virus^a*^	Bacteria^b*^	Protozoa^c*^	No. of microparasite infections^†^
Any STH^d^	0.48 (0.28, 0.83)	1.81 (1.06, 3.07)	1.50 (0.95,2.37)	0.11 (0.01, 0.21)
Hookworm^e^	0.70 (0.40, 1.21)	1.22 (0.70, 2.12)	1.78 (1.11, 2.84)	0.09 (0.00, 0.19)
*A. lumbricoides*	0.66 (0.23, 1.87)	1.02 (0.35, 2.96)	1.42 (0.59, 3.41)	0.01 (−0.16, 0.18)
*T. trichiura*	0.53 (0.22, 1.29)	5.97 (2.05, 17.4)	1.79 (0.84, 3.80)	0.18 (0.03, 0.33)
*S. stercoralis*	0.52 (0.29, 0.95)	1.32 (0.69, 2.53)	1.30 (0.78, 2.17)	0.08 (−0.02, 0.18)

^a^Virus includes one or more of the following pathogens: astrovirus, adenovirus, norovirus GI, norovirus GII, rotavirus, or sapovirus

^b^Bacteria includes one or more of the following pathogens: *Aeromonas*, *C. difficile*, *C. jejuni*, EAEC, EHEC, EPEC (typical or atypical), LT- or ST-ETEC, *Shigella* spp. enteroinvasive *E. coli*, or *Salmonella*

^c^Protozoa includes one or more of the following pathogens: non-hominus and non-parvum *Cryptosporidium* spp., *C. hominus*, *C. parvum*, *E. histolytica*, and *G. intestinalis*

^d^Any soil-transmitted helminth (STH) includes one or more of the following helminths: hookworm (*N. americanus* and/or *A. duodenale*)*, A. lumbricoides*, *T. trichiura*, or *S. stercoralis*

^e^Hookworm includes *N. americanus* and/or *A. duodenale*

* Results are adjusted odds ratios and 95% confidence intervals and are interpreted as the change in odds of virus/bacteria/protozoa infection among subjects with STH (or specific STH species) infection compared to those without

^†^Results are beta coefficients and 95% confidence intervals and are interpreted as the change in number of microparasite infections among subjects with STH (or specific STH species) infection compared to those without

*Notes*: All models control for population group, sex, socioeconomic status, ethnic minority status, household population size, presence of an improved toilet in household, use of an improved household drinking water source, presence of soap for handwashing at household, household animal ownership, and whether the village school was a beneficiary of the UNICEF WASH in Schools intervention, and include random intercepts at the village and household level

STH infections were associated with a higher number of total concurrent microparasite infections (Table 3). This trend was consistent across all STH species, and was statistically significant for any STH infection (change in number of microparasite infections among subjects with STH infection compared to those without (*β*)= 0.11, 95% CI: 0.01–0.21) and *T. trichiura* (*β *= 0.18, 95% CI: 0.03–0.33).

## Discussion

Within-host interactions between helminths and microparasites can affect a range of factors, including whether a pathogen can establish itself in a host, rate of growth and replication within a host, rate of clearance from the host, and severity of disease [19]. Evidence supporting whether such co-infections result in beneficial, harmful, or neutral interactions is mixed [3, 4, 18], and the mechanisms by which helminths and microparasites interact are not clearly established [11, 18]. Most studies of co-infection have examined interactions between two species [16], often utilizing *in vitro* or animal models and/or employing helminths and microparasites that are not commonly found in humans [12, 32–37]. Our approach addresses the limitations of these previous studies by taking a macro approach to co-infection in humans. Rather than examining pairwise associations between pathogens, we enhance our generalizability by examining the associations between STH and microparasite taxa. Additionally, we control for the presence of other pathogen taxa beyond those of immediate interest, which is more realistic for low-income settings where humans harbor multiple infections that may have antagonistic or synergistic interactive effects [2–4]. Our analysis revealed a clear trend in which STH infection was associated with reduced odds of concurrent viral infection and increased odds of concurrent bacterial infection. STH infection was also associated with increased odds of protozoal infection, although this association was statistically significant only for the most prevalent STH, hookworm.

Our results are consistent with previous research reporting that helminths impair host immunity to concurrent enteric bacterial infection [7, 37, 38]. Helminth infection causes intestinal barrier dysfunction and increased “leakiness” of the intestinal epithelium [37, 39], which is one mechanism by which STH infection may increase odds of concurrent bacterial infection. For many enterobacteria to infect a host, the pathogen must exit the intestinal lumen and cross the epithelial barrier to invade cells in the small and large intestine [37, 40]. Intestinal epithelial cells are critical for gut homeostasis because they form physical and chemical barriers that protect the intestinal epithelia from invading pathogens [40]. For example, the Ly6/Plaur domain-containing 8 (Lypd8) protein, which is a physical barrier found in the uppermost epithelial layer of the large intestine, inhibits invasion of the colonic epithelia by bacteria in the genera *Escherichia*, *Proteus* and *Helicobacter* [41]. Antimicrobial peptides (AMPs) are chemical barriers found in the small intestine that include defensin proteins, which cause cell disruption and protect against pathogenic bacterial invasions such as *S. typhimurium* [40, 42]. Therefore, the enhanced permeability of the intestinal barrier due to helminth infection may facilitate the penetration of bacterial endotoxins [39, 43]. Further, hosts rely on their innate immune system to respond to such attacks through activation of Toll-like receptors, secretion of chemoattractant molecules and cytokines, and recruitment of cells such as neutrophils, monocytes, dendritic cells and lymphocytes [37]. However, helminths can modulate this innate immune response to bacterial enteropathogens by stimulating regulatory cytokines (such as IL-10), antagonizing proinflammatory factors that can lead to more severe intestinal inflammation (such as keratinocyte-derived chemokine and macrophage inflammatory protein 2), impeding clearance of pathogens, and reducing availability of pathogen-specific cytokines [11, 37, 43].

We also found that STH infection, specifically hookworm, was associated with increased odds of concurrent protozoal infection. Our results are consistent with previous research in Venezuela, which found that *Giardia* prevalence was significantly higher among children harboring an *A. lumbricoides* infection compared to those without [44]. We found that protozoal infections were driven largely by *Giardia*, as 94.7% of subjects with a protozoal infection had *Giardia*. One possible mechanism by which helminths may increase susceptibility to protozoa is through the proinflammatory cytokine IFN-γ [45, 46], which is antagonized by the cytokine IL-4 triggered by helminth infection [15]. Evidence suggests that IFN-γ is significantly higher among humans infected with *Giardia* and *E. histolytica*, suggesting this cytokine has a protective role in host defense [46–48]. However, helminths suppress IFN-γ, which may impede the host from mounting an effective immune response [18]. Additionally, intestinal barrier dysfunction and increased permeability of the intestinal lumen caused by helminth infection may be exacerbated by protozoal infection, thus facilitating the translocation of antigens and inducing a pro-inflammatory response within the intestine [46]. It is also possible that increased odds of STH infection given protozoal infection is reflecting the inverse association; in other words, that protozoal infection increases the odds of STH infection. *Giardia* is one of the earliest infections that children succumb to [31, 49], and can result in chronic infection [49, 50]. Like helminths, *Giardia* immunomodulates the host immune system and causes gut dysfunction [51, 52]. Thus, it is possible that chronic *Giardia* infection early in life may have preceded and enhanced susceptibility to STH infection.

Helminths are generally thought to increase transmission, virulence and progression of microparasite infection, and reduce recovery [4, 15, 17], as supported by our results for bacterial and protozoal infections. However, some exceptions have been established in the literature [35, 53–55], and helminths are being explored as a possible curative tool for immune-mediated conditions such as allergies, asthma and ulcerative colitis [56–58]. We found that helminth infection was negatively associated with odds of concurrent viral infection, contradicting existing research indicating that helminths may limit both innate and adaptive immune responses to viral infection [36, 59]. However, it is possible that helminths are protective against viral infection because the Th2 immune response induced by helminth infection has anti-inflammatory and wound-healing properties [11, 15]. In the present study, viral infections were driven largely by rotavirus (60.5% of subjects with a viral infection), followed by norovirus GII (22.2%). Rotavirus infection induces oxidative stress and inflammatory signaling; this pro-inflammatory signaling is necessary for virus replication, but is inhibited by anti-inflammatory treatment [60]. Norovirus infection also causes alterations of the gut mucosa, including mucosal inflammation [61]. When a microparasite such as rotavirus or norovirus induces inflammation-mediated damage, helminths may protect the host from damage by secreting IL-10 and TGF-β and decreasing the production of pro-inflammatory cytokines [62], which may be protective against the detrimental inflammatory Th1 response induced by viral microorganisms [11, 19, 35, 62].

### Strengths and limitations

Strengths of this study include the random selection of participating villages and households. Also, pathogens in stool samples were detected and quantified using qPCR, which provides a higher sensitivity (98%) and specificity (100%) than conventional methods [24]. Further, the multi-target detection capacity of this method allowed us to examine 25 infectious pathogens [24], whereas most existing studies on pathogen co-infection have focused on pairs of agents [16]. Additionally, there is a dearth of clinical data on helminth co-infection, and most studies have relied on mouse models [35]. The predominant species involved in human STH and enteric microparasite infection is influenced by a range of factors, including age and WASH access [63, 64]. We examined human subjects from three distinct age groups, adults, school-aged children and children under five years-old, and controlled for potential confounding WASH and demographic variables to provide a more externally valid picture of STH and microparasite co-infection.

Our study is subject to a number of limitations. First, our data are cross-sectional so we do not know whether the STH or microparasite infection occurred first. However, STH are endemic in this population, our data were collected prior to annual primary school-based PC, there is no routine community-based PC in this population, and re-infection often occurs rapidly after PC [65]. Thus, it is likely that STH infections commonly preceded microparasite infections, particularly viral and bacterial infections which do not tend to be chronic. However, it is possible that some persistent protozoal infections (e.g. *Giardia*) preceded STH infection, and our results reflect an inverse association. Secondly, the high sensitivity of the TAC and other molecular assays may lead to the detection of prolonged shedding by attenuated pathogens and we cannot distinguish between symptomatic and asymptomatic infections [66]. However, even asymptomatic infections may lead to interactions within the host as well as other sequalae such as environmental enteropathy, malnutrition, and growth stunting [67–69]. Thirdly, evidence suggests that the outcomes of helminth-microparasite co-infection are context dependent and may depend on helminth infection intensity [19, 44]. Based on the Kato-Katz results from these samples, helminth infections were predominately of low infection intensity, so we were unable to stratify by infection intensity to evaluate differences in co-infection by infection intensity. Fourthly, we discarded 144 samples due to suspected contamination, which may have limited statistical power. Household toilet ownership and use of an improved water source were lower among participants whose samples were discarded. While these factors may be associated with the pathogen profile of the participants, contamination was a random event unassociated with the participants and would not confound the relationship between STH infection and odds of microparasite infection. Last, we did not measure cytokines, interferon, or other measures of immune response so we are unable to elucidate exact mechanisms of helminth-microparasite interaction.

## Conclusions

The effects of helminth infection on odds of concurrent microparasite infection differed by microparasite taxa. We found that helminth infection was negatively associated with concurrent viral infection, but positively associated with concurrent bacterial and protozoal infections, after controlling for shared risk factors for infection. These results suggest that interventions to control STH, such as increasing community sanitation coverage to eliminate the environmental reservoir for STH, combined with PC with anti-helminthic drugs [70, 71], could have a spillover impact on bacterial and protozoal infections. Increased integration and collaboration between WASH and STH sectors is warranted [71]. Additional research is needed to elucidate the exact mechanism of immunomodulatory effects of STH on concurrent enteric microparasite infection.

## Additional files

**Additional file 1: Table S1.** Primers and probes for custom TaqMan Array Card.

**Additional file 2: Figure S1.** Soil-transmitted helminth infection intensity according to Kato-Katz test, Saravane Province, Laos, 2017 (*n *= 746).

## Abbreviations

AMP: antimicrobial peptide
BSA: bovine serum albumin
CI: confidence interval
EAEC: enteroaggregative *E. coli*
EHEC: enterohemorrhagic *E. coli*
EIEC: enteroinvasive *E. coli*
EPEC: enteropathogenic *E. coli*
ETEC: enterotoxigenic *E. coli*
IFN: interferon
IL: interleukin
JMP: Joint Monitoring Programme
Lao PDR: Laos: Lao People’s Democratic Republic
Lypd8: Ly6/Plaur domain-containing 8 (protein)
OR: odds ratio
PC: preventative chemotherapy
qRT-PCR: quantitative reverse transcription polymerase chain reaction
STH: soil-transmitted helminth
TAC: TaqMan Array Card
TGF: transforming growth factor
Th1: Type 1 (immune response)
Th2: Type 2 (immune response)
TNF: tumor necrosis factor
T_reg_: regulatory T cell
WASH: water, sanitation, and hygiene
WASH HELPS: Water, Sanitation, and Hygiene for Health and Education in Laotian Primary Schools
WHO/UNICEF: World Health Organization/United Nations International Children’s Fund
