# Supplementary material for: Associations between soil-transmitted helminthiasis and viral, bacterial, and protozoal enteroinfections: a cross-sectional study in rural Laos
Source: Parasit Vectors. 2019 May 7;12:216. doi: 10.1186/s13071-019-3471-2 (PMC6505259; doi:10.1186/s13071-019-3471-2)
Supplement: Supplementary file 2 — Additional file 2: Figure S1. Soil-transmitted helminth infection intensity according to Kato-Katz test, Saravane Province, Laos, 2017 (n = 746). [file 13071_2019_3471_MOESM2_ESM.docx]

Additional file 2: Figure S1. Soil-transmitted helminth infection intensity according to Kato-Katz test, Saravane Province, Laos, 2017 (n=746)

**
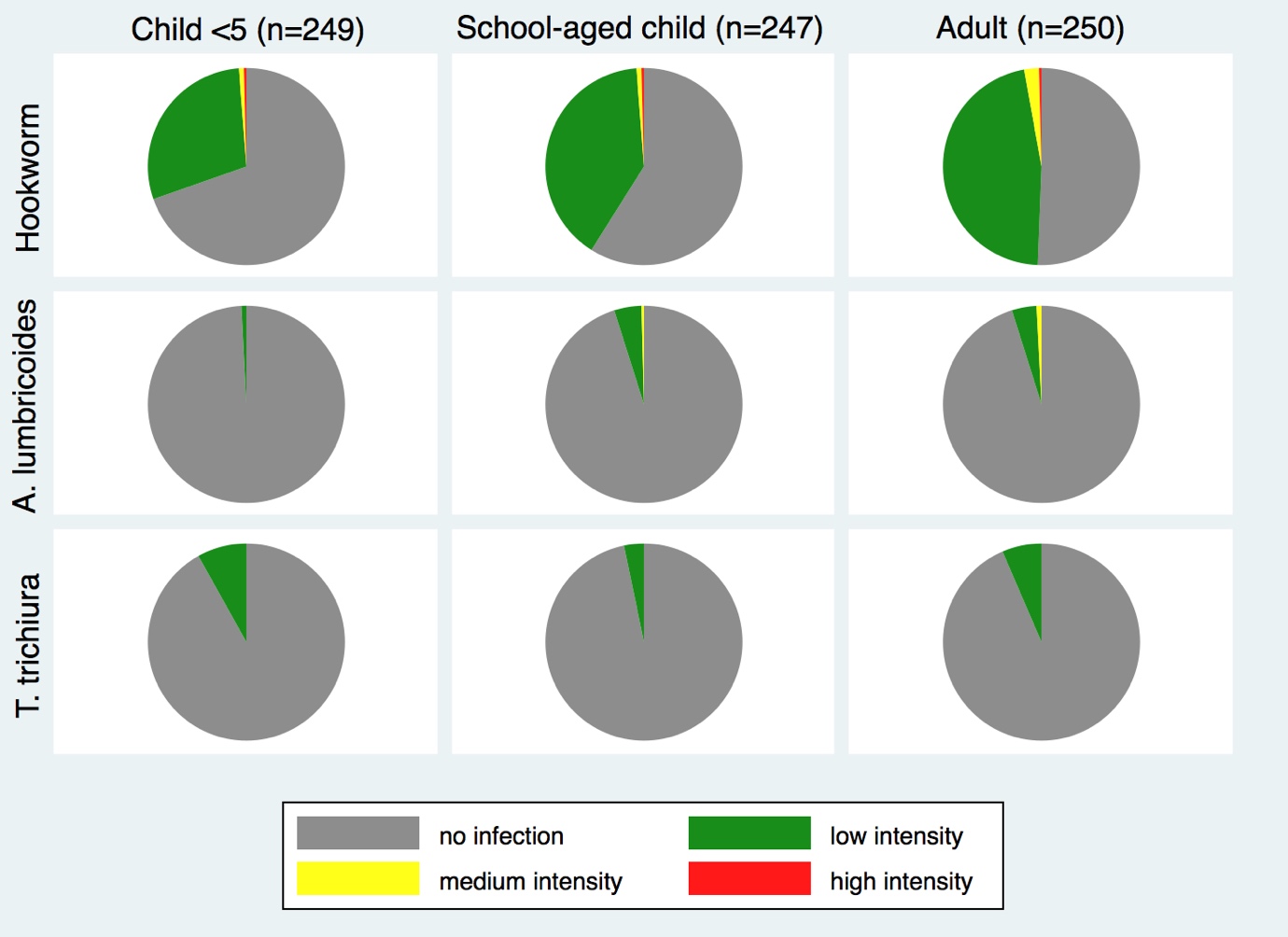
**
